# Scoring system for predicting 90-day mortality of in-hospital liver cirrhosis patients at Cipto Mangunkusumo Hospital

**DOI:** 10.1186/s12876-023-02813-4

**Published:** 2023-06-01

**Authors:** Irsan Hasan, Saut Horas Hatoguan Nababan, Anugrah Dwi Handayu, Gita Aprilicia, Rino Alvani Gani

**Affiliations:** Hepatobiliary Division, Department of Internal Medicine, Faculty of Medicine Universitas Indonesia, Cipto Mangunkusumo National General Hospital, Jakarta, Indonesia

**Keywords:** Scoring system, Liver cirrhosis, Mortality

## Abstract

**Background:**

Liver cirrhosis is the final stage of chronic liver disease. Complications due to progression of liver disease may deteriorate the liver function and worsen prognosis. Previous studies have shown that patients with liver cirrhosis are at increased risk of death within 90-day after hospitalization. It is necessary to identify patients who are at higher risk of early mortality. This study aims to develop a scoring system to predict the 90-day mortality among hospitalized patients with liver cirrhosis that could be used for modification of treatment plan according to the scores that have been obtained. By using this scoring system, crucial care of plans can be taken to reduce the risk of mortality.

**Method:**

This prospective cohort study was conducted on hospitalized cirrhotic patients at Cipto Mangunkusumo National General Hospital, Jakarta. Demographic, clinical, and laboratory data were recorded. Patients were monitored for up to 90-day after hospitalization to determine their condition. Cox regression analysis was performed to obtain predictor factors contributing to mortality in liver cirrhosis patients. The scoring system that resulted from this study categorized patients into low, moderate, and high-risk categories based on their predicted mortality rates. The sensitivity and specificity of the scoring system were evaluated using the AUC (area under the curve) metric.

**Result:**

The study revealed that liver cirrhosis patients who were hospitalized had a 90-day mortality rate of 42.2%, with contributing factors including Child-Pugh, MELD, and leukocyte levels. The combination of these variables had a good discriminative value with an AUC of 0.921 (95% CI: 0.876–0.967). The scoring system resulted in three risk categories: low risk (score of 0–3) with a 4.1-18.4% probability of death, moderate risk (score of 5–6) with a 40.5-54.2% probability of death, and high risk (score of 8–11) with a 78.1-94.9% probability of death.

**Conclusion:**

The scoring system has shown great accuracy in predicting 90-day mortality in hospitalized cirrhosis patients, making it a valuable tool for identifying the necessary care and interventions needed for these patients upon admission.

## Background

The mortality rate of patients with liver cirrhosis has significantly increased in recent years, including in Indonesia. Based on research conducted by Mokdad et al. in 1980–2010, Indonesia ranked fourth in the world for the highest mortality rate of cirrhotic patients compared to 186 other countries [1]. According to the Institute for Health Metrics and Evaluation (IHME), during the period of 2007–2017, liver cirrhosis increased by 5.6% as the most common cause of mortality in Indonesia [2]. In 2016, a study by Gani et al. at our hospital showed that the mortality rate of patients with liver cirrhosis in the first 2 years was 75.3%, with the highest mortality occurring in the first 3 months after hospital admission [3]. A retrospective cohort study by Gani et al. using medical record data of hospitalized patients in 2017 found that the 90-day mortality rate in hospitalized patients was 54.8%, where the contributing factors included age, leukocytes, and Child-Pugh [4].

Multiple factors, including intrahepatic and extrahepatic factors, can cause death in liver cirrhosis patients [5] A previous study conducted at our hospital showed that infection was the most common cause of death in these patients [6]. The high mortality rate in patients with liver cirrhosis has raised particular concerns for clinicians to develop a method to accurately identify patients at high-risk so that treatments can be modified to reduce the risk of death.

The most widely used predictors of mortality in patients with liver cirrhosis (LC) worldwide are the Child-Pugh Score (CP) and the Model for End-stage Liver Disease (MELD) [7–9]. In the USA, a study conducted by Kartoun et al. introduced MELD-Plus (an improvement of MELD and MELD-Na scores) to predict the mortality rate of patients with liver cirrhosis in the first 90-day after hospital admission [10]. In Indonesia, Gani et al. assessed the prognosis predictor of mortality in LC patients using Child-Pugh and MELD scoring system. The results showed that CP scores > 7 and MELD > 9 could be good predictors with sensitivity and specificity up to 70% [3]. However, no studies in Indonesia specifically discuss the scoring system to assess 90-day mortality after hospital admission in liver cirrhosis patients. Thus, the aim of this study is to develop a scoring system to predict the mortality rate of patients with liver cirrhosis in the first three months after hospital admission at Cipto Mangunkusumo General Hospital, Jakarta.

## Methods

### Study population and design

The prospective cohort study was conducted at Cipto Mangunkusumo General National Hospital (RSCM) from 2020 to 2021. The data collected included the clinical characteristics of cirrhotic patients during hospital admission, 90-day survival after hospitalization, and the patient’s cause of death. This study has been approved by the Ethics Committee of the Faculty of Medicine, University of Indonesia.

### Inclusion and exclusion criteria

The population of this study consisted of hospitalized patients with liver cirrhosis at Cipto Mangunkusumo General Hospital. The inclusion criteria were: (a) patients with liver cirrhosis confirmed by ultrasonography or fibroscan, (b) adults aged above 18 years (c) admitted to the hospital for the first time with emergency causes or complications from cirrhosis, (d) willing to participate in the study by signing the informed consent form, (e) underwent anamnesis and had 100 ml blood taken for laboratory examination, (f) able to be followed up at least 3 months after hospitalization. The exclusion criteria were: (a) admitted for elective procedures such as biopsy, endoscopy, and ligation, (b) presence of hepatocellular carcinoma, malignancy, or HIV AIDS, (c) history of a liver transplant, e) special or vulnerable patients such as prisoners or homeless individuals.

### Data collection and analysis

The data were analyzed using SPSS. Basic characteristic such as gender, age, etiology, Child-Pugh, and MELD categories were described as a proportion if the data were categorical and described as mean or median if the data was numerical. Clinical characteristics and liver function as factors affecting 90-day mortality in liver cirrhosis patients after hospitalization were tested using the Multivariate Cox Regression test. A p-value below 0.05 was declared statistically significant. The scoring system was assessed to develop the score for each predictor variables contributing in 90-day of mortality. To avoid the occurrence of intercorrelation among two or more independent variables, multicollinearity was assessed by examining tolerance and the Variance Inflation Factor (VIF). The score was calculated using the beta coefficient and standard error. The final scores were classified based on the risk of patients with high, moderate, and low mortality rates. Based on the scoring results, sensitivity and specificity were assessed using AUC (under curved area).

## Results

**Fig. 1:**
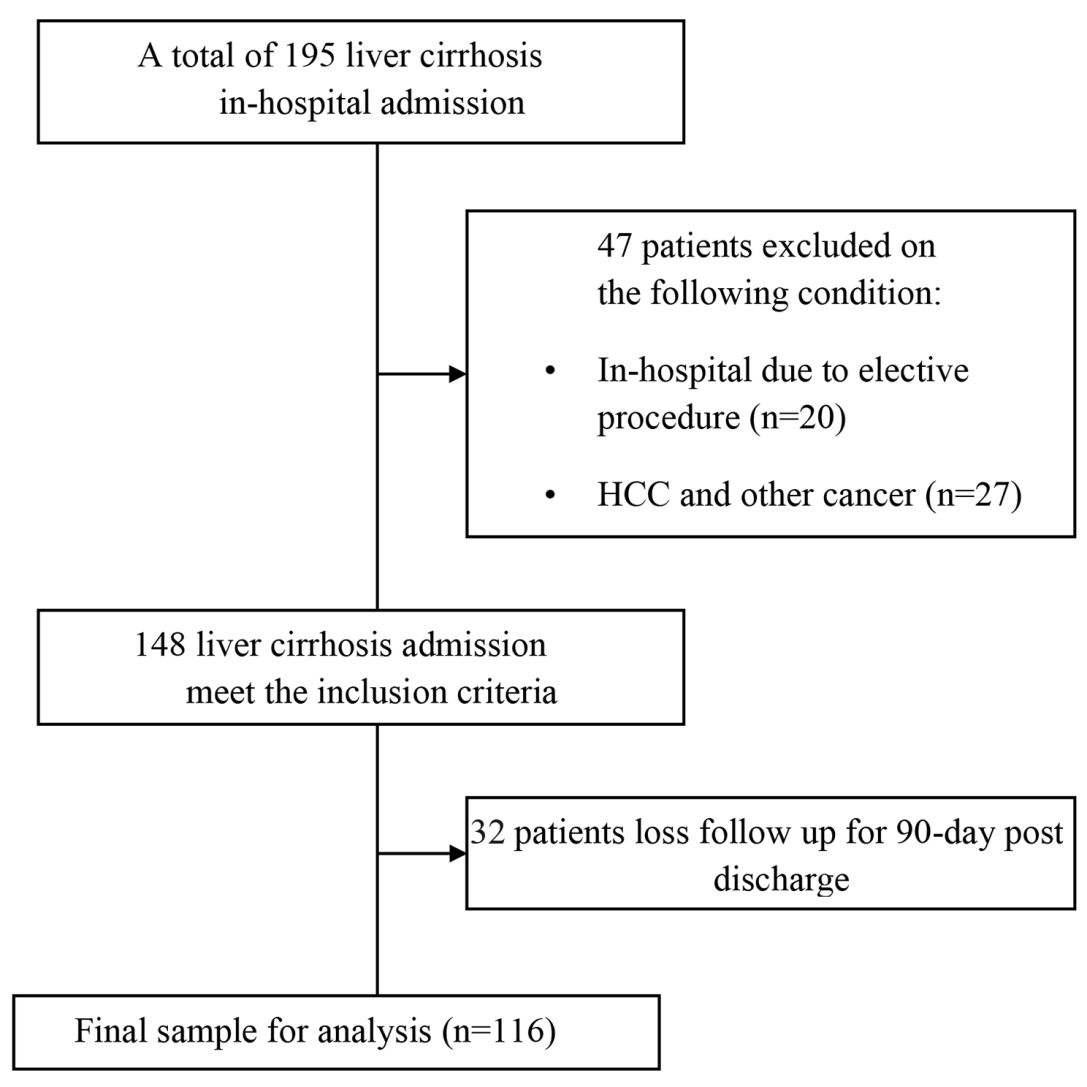
Flow Chart of Subject Selection

One hundred and ninety-five patients with liver cirrhosis were hospitalized from 2020 to 2021. However, 47 patients were excluded due to elective procedures (n = 20) and malignancy (n = 27). Out of the remaining 148 patients with liver cirrhosis who were admitted to the hospital, 32 patients were loss to follow-up and could not be reached within 90-day post-discharge. Thus, the final analysis included 116 patients, as shown in Fig. 1.

The characteristics of the subjects were shown in Table 1. The mean age of patients with liver cirrhosis at hospital admission was 54 years (standard deviation ± 13.01), and 41 patients (35.3%) were elders above 60 years. Gender was dominated by men (84/116 patients; 72.4%). The most common etiology found in patients with liver cirrhosis was Non-B Non-C (NBNC). Etiological of NBNC liver cirrhosis were metabolic syndrome (20.7%), primary biliary cirrhosis (4.3%), alcoholic liver disease (3.4%), autoimmune (1.7%), and unspecified NBNC (6%). LC patients were dominated by Child-Pugh C (42.2%). A total of 56.0% patients had MELD above 14. The median AST value was 53 (12-1927) U/L and the median ALT value was 32 (6-509) U/L. The median length of stay (LOS) in the hospital was 6 (1–34) days. The median LOS of deceased patients was not significantly longer than survived patients (7 (1–34) days vs. 6 (1–26) days, respectively). There were 2.6% of patients hospitalized within 30 days. Ascites massive has been the leading cause of hospitalization in LC patients (37.9%), followed by variceal bleeding (36.2%), hepatic encephalopathy (21.6%), and jaundice (24.1%). A total of 42.2% subjects died at 90-day of in-hospital observation. The causees of death were shown in Table 2. Among our LC patients, the cause of death varied, with the most common causes being hepatic encephalopathy (40.8%), late onset HAP (34.7%), renal failure (22.4%), respiratory failure (20.4%), septic shock (18.4%), SBP (12.2%), hepatic failure (8.2%). The cause of death was obtained from the death certificate and electronic health medical record in the hospital using the ICD-10 coding system.

**Table 1:** Characteristics of Subjects

Variables	All Subjects (n = 116)	Survive (n = 67)	Died (n = 49)	P value
Age, mean ± SD	54 ± 13.01	53 ± 12.48	55 ± 13.69	0.306
Age, n (%)				
< 60 years	75 (64.7)	45 (67.2)	30 (61.2)	-
≥ 60 years	41 (35.3)	22 (32.8)	19 (38.8)	0.662
Sex, n (%)				
Female	32 (27.6)	18 (26.9)	14 (28.6)	-
Male	84 (72.4)	49 (73.1)	35 (71.4)	0.971
**Comorbidity**				
Diabetes Mellitus, n (%)				
No	83 (71.6)	46 (68.7)	37 (75.5)	-
Yes	33 (28.4)	21 (31.3)	12 (24.5)	0.549
Hypertension, n (%)				
No	100 (86.2)	56 (83.6)	44 (89.8)	-
Yes	16 (13.8)	11 (16.4)	5 (10.2)	0.493
Acute Kidney Injury, n (%)				
No	110 (94.8)	63 (94.0)	47 (95.9)	-
Yes	6 (5.2)	4 (6.0)	2 (4.1)	0.977
Chronic Kidney Disease, n (%)				
No	110 (94.8)	64 (95.5)	46 (93.9)	-
Yes	6 (5.2)	3 (4.5)	3 (6.1)	1.000
Stroke, n (%)				
No	113 (97.4)	65 (97.0)	48 (98.0)	-
Yes	3 (2.6)	2 (3.0)	1 (2.0)	1.000
Etiology, n (%)				
Unspecified NBNC	7 (6)	4 (6)	3 (6.1)	-
Autoimmune	2 (1.7)	1 (1.5)	1 (2)	0.858
Alcoholic	4 (3.4)	3 (4.5)	1 (2)	0.558
MAFLD	24 (20.7)	17 (25.4)	7 (14.3)	0.499
NASH	2 (1.7)	2 (3.0)	0 (0)	0.999
Primary biliary cirrhosis	5 (4.3)	1 (1.5)	4 (8.2)	0.216
HBV	39 (33.6)	24 (35.8)	15 (30.6)	0.826
HCV	33 (28.4)	15 (22.4)	18 (36.7)	0.576
Child-Pugh score, mean ± SD	8 ± 2.73	7 ± 2.05	11 ± 2.05	< 0.001
Child-Pugh class, n (%)				
A	31 (26.7)	30 (44.8)	1 (2.0)	-
B	36 (31.0)	26 (38.8)	10 (20.4)	0.027
C	49 (42.2)	11 (16.4)	38 (77.6)	< 0.001
MELD score, mean ± SD	18 ± 9.73	12 ± 5.09	26 ± 8.69	< 0.001
MELD, n (%)				
< 14	51 (44.0)	46 (68.7)	5 (10.2)	-
≥ 14	65 (56.0)	21 (31.3)	44 (89.8)	< 0.001
AST, U/L, median (min-max)	53 (12–1927)	43 (12–226)	72 (25–1927)	< 0.001
ALT, U/L, median (min-max)	32 (6–509)	26 (9–121)	39 (6–509)	0.049
Hemoglobin, g/dl, mean ± SD	9.53 ± 2.15	9.82 ± 2.20	9.12 ± 2.03	0.084
Platelet, x 10^3^/mm^3^, mean ± SD	110.1 ± 70.83	106 ± 63.07	114 ± 80.71	0.541
Leukocytes, x 10^3^/mm^3^, median (min-max)	8.1 (1.2–55.9)	5.1 (1.2–39.0)	15.1 (1.6–55.9)	< 0.001
Leukocytes, n (%)				
< 12.000	81 (69.8)	61 (91)	20 (40.8)	-
≥ 12.000	35 (30.2)	6 (9)	29 (59.2)	< 0.001
Albumin	2.66 (1.22–4.17)	2.94 (1.79–4.17)	2.43 (1.22–3.44)	< 0.001
Bilirubin	2.16 (0.36–38.36)	2.1 (0.4–15.6)	6.6 (0.4–38.4)	< 0.001
Protrombin time	13.4 (10.6–120.0)	12.7 (10.6–16.5)	15.9 (10.9–120.0)	< 0.001
INR	1.26 (0.96–11.76)	1.21 (0.96–1.53)	1.47 (1.04–11.76)	< 0.001
Ureum	48.6 (9.6–458)	31 (9.6–162)	83 (24–458)	< 0.001
Creatinine	0.9 (0.28–8.10)	0.9 (0.4–3.6)	1.2 (0.3–8.1)	< 0.001
Length of stay	6 (1–34) days	6 (1–26) days	7 (1–34) days	0.586
Hospitalized within 30 days, n (%)				
No	113 (97.4)	67 (100)	46 (93.6)	-
Yes	3 (2.6)	0 (0)	3 (6.1)	0.073
Cause of first hospitalization				
Variceal bleeding, n (%)				
No	74 (63.8)	43 (64.2)	31 (63.3)	-
Yes	42 (36.2)	24 (35.8)	18 (36.7)	1.000
Jaundice, n (%)				
No	88 (75.9)	62 (92.5)	26 (53.1)	-
Yes	28 (24.1)	5 (7.5)	23 (46.9)	< 0.001
Hepatic encephalopathy, n (%)				
No	72 (62.1)	54 (80.6)	18 (36.7)	-
Grade 1–2	19 (16.4)	7 (10.4)	12 (24.5)	0.003
Grade 3–4	25 (21.6)	6 (9.0)	19 (38.8)	< 0.001
Ascites, n (%)				
No	66 (56.9)	50 (74.6)	16 (32.7)	-
Mild	6 (5.2)	4 (6.0)	2 (4.1)	0.625
Massive	44 (37.9)	13 (19.4)	31 (63.3)	< 0.001

**Table 2:** Cause of death in deceased patients

Cause of death in 49 LC patients, n (%)	
Septicaemia	9 (18.4)
Septic shock	9 (18.4)
Hemorrhagic shock	2 (4.1)
Hypovolemic shock	2 (4.1)
Cardiogenic shock	2 (4.1)
Renal failure	11 (22.4)
Respiratory failure	10 (20.4)
Hepatic failure	4 (8.2)
Hepatic encephalopathy	20 (40.8)
HAP late onset	17 (34.7)
SBP	6 (12.2)

**Fig. 2:**
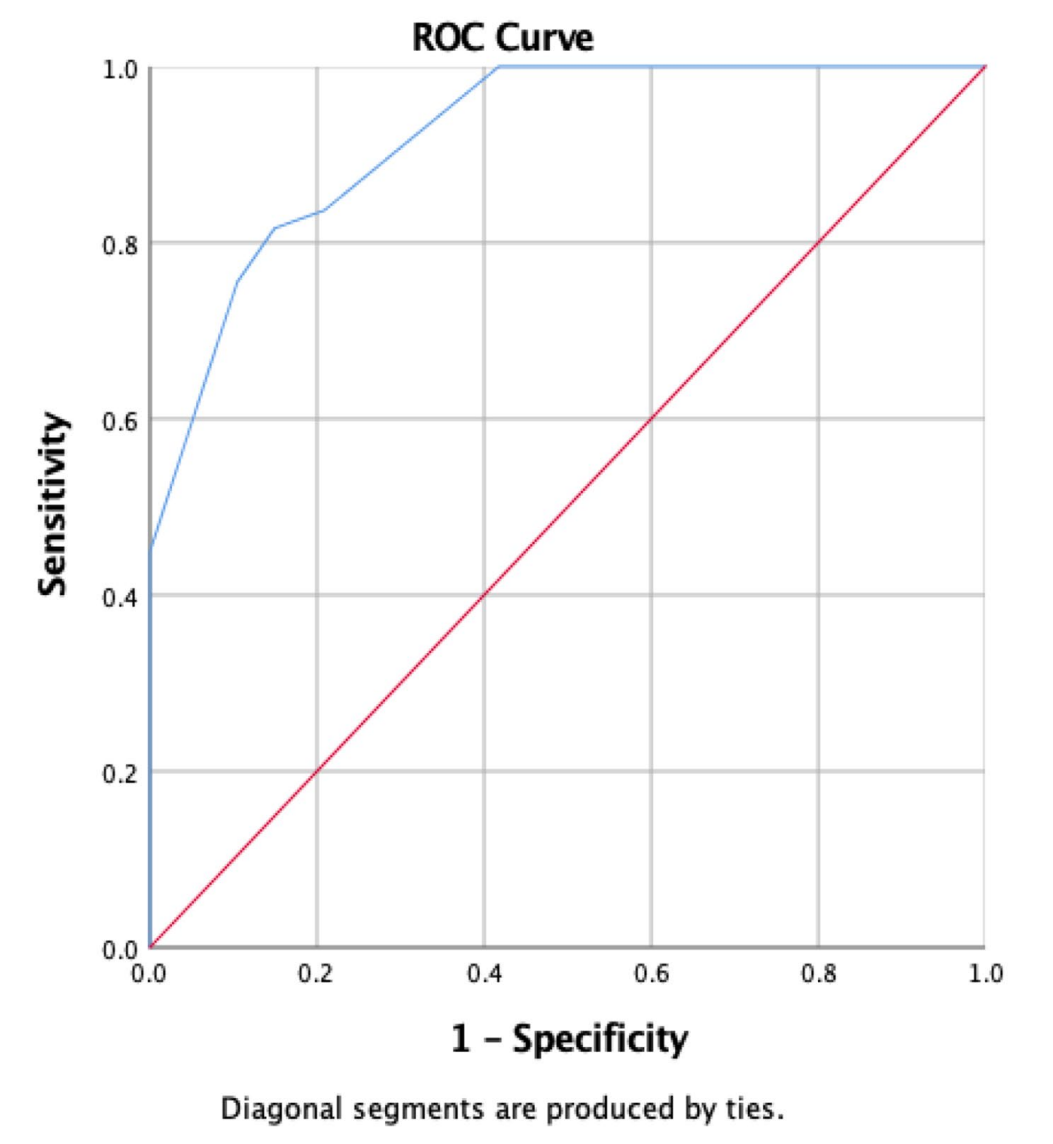
AUC 90-Day Mortality Patients with Liver Cirrhosis Post Hospitalization

The multivariate analysis showed that Child-Pugh, MELD and leukocytes were statistically significant predictors of 90-day mortality of LC patients after hospitalization at Cipto Mangunkusumo General Hospital, as summarized in Table 3. Table 4 showed that the combination of Child-Pugh, MELD, and Leukocytes provided the best discrimination value based on the AUC (0.921; 95% CI: 0.876–0.967), with a sensitivity of 81.6% and a specificity of 85.1%. These results are presented in Fig. 2. Each of independent factors in multivariate analysis was assessed to create calculation for 90-day mortality scoring system (Table 5). The scoring system for predicting 90-day mortality based on the significant variables was presented in Table 6, and its clinical application was demonstrated in Table 7. An increase in scores suggests that liver cirrhosis patients have a higher likelihood of experiencing a negative outcome.

**Table 3:** Predictors of Mortality of Liver Cirrhosis Patients in 90-Day Post Hospitalization at Cipto Mangunkusumo Hospital

Variables	Bivariate		Multivariate	
	HR (95% CI)	p	HR (95% CI)	p
Age, n (%)				
< 60 years	1	-		
≥ 60 years	1.14 (0.64–2.02)	0.662		
Sex, n (%)				
Female	1	-		
Male	1.01 (0.54–1.88)	0.971		
Etiology, n (%)				
NBNC	1	-		
HBV	1.09 (0.54– 2.20)	0.812		
HCV	1.71 (0.89–3.35)	0.120		
Child-Pugh, n (%)				
A	1	-	1	-
B	10.14 (1.30–79.21)	0.027	4.97 (0.59–41.38)	0.138
C	44.21 (6.05–323.22)	< 0.001	14.59 (1.77–120.16)	0.013
MELD, n (%)				
< 14	1	-	1	-
≥ 14	11.16 (4.41–25.25)	< 0.001	3.20 (1.16–8.85)	0.001
Leukocytes, n (%)				
< 12.000	1	-	1	-
≥ 12.000	5.11 (2.86–9.14)	< 0.001	2.83 (1.54–5.17)	0.001

**Table 4:** Predicted Value of Prognostic Parameters of Mortality of Patients with Liver Cirrhosis in 90-Day

Variable	AUC	IK 95%	Cut off	Sensitivity	Specificity	p
Child-Pugh	0.817	0.738–0.897	9	91.8%	71.6%	< 0.001
MELD	0.792	0.708–0.876	14	89.8%	68.7%	< 0.001
Leukocytes	0.751	0.656–0.846	12.000	59.2%	99.1%	< 0.001
Child-Pugh + MELD + Leukocytes	0.921	0.876–0.967	5	81.6%	85.1%	< 0.001

**Table 5:** Assessment of the Score Value of Predictors Factor

Variable	B	SE	B/SE	Score
Child-Pugh	2.769	0.577	4.79	5
MELD	1.192	0.423	2.81	3
Leukocytes	1.032	0.312	3.30	3

**Table 6:** Scoring System for Prediction 90-Day Mortality in Cirrhosis Patients

No	Scoring Parameters	Yes	No	Total Score
1	Is the patient with liver cirrhosis admitted to the hospital in a state of Child-Pugh C?	5	0	
2	Is the patient’s MELD score above 14 when the patient with liver cirrhosis is hospitalized?	3	0	
3	Is the patient’s leukocyte above 12.ooo when the patient with liver cirrhosis is hospitalized?	3	0	

**Table 7:** Clinical Application of Scoring System 90-Day Mortality in Cirrhosis Patients

Score	Mortality Risk (%)	Sensitivity (%)	Specificity (%)
0	4.1%	100%	58.2%
3	18.4%	83.7%	79.1%
5	40.5%	81.6%	85.1%
6	54.2%	75.5%	89.6%
8	78.1%	44.9%	100%
11	94.9%	0%	100%

**Table 7:** Clinical Application of Scoring System 90 Days Mortality in Cirrhosis Patients

Score	Mortality Risk (%)	Sensitivity (%)	Specificity (%)
0	4.1%	100%	58.2%
3	18.4%	83.7%	79.1%
5	40.5%	81.6%	85.1%
6	54.2%	75.5%	89.6%
8	78.1%	44.9%	100%
11	94.9%	0%	100%

## Discussion

### 90-Day mortality of patients with liver cirrhosis after hospitalization

The LC patients in this study were dominated by men (72.4%) with a mean age of 54 years. In Indonesia, cirrhosis is typically caused by viral hepatitis such as HBV and HCV infection. However, in our cohort, there has been a significant increase in the incidence of non-viral cirrhosis, such as MAFLD and NASH. These conditions are strongly linked to metabolic syndrome, which includes obesity, hypertension, diabetes mellitus, and dyslipidemia, and are becoming increasingly recognized as important causes of cirrhosis worldwide, including in Indonesia. The etiology of non-B, non-C (NBNC) cirrhosis caused by alcohol consumption is not commonly observed in Indonesia. This may be due to the high prices of alcoholic beverages and cultural factors that discourage alcohol consumption in the country.

The 90-day mortality rate of liver cirrhosis patients after being hospitalized at Cipto Mangunkusumo Hospital in this study was 42.2%. Gani et al. carried out a research on patients with liver cirrhosis at similar center between 2011 and 2016. The study findings revealed a two-year survival rate after hospitalization was 24.7%, and 50% of patients died within five months of observation [3]. A retrospective cohort study by Gani et al. in 2017 showed that 90-day mortality rate was around 13% higher than in our prospective cohort [4]. The different compositions of cirrhotic patients in our cohort could explain this finding. In the previous cohort, LC patients with HCC and other malignancies were included as study participants, which contributed to the high mortality rate. However, the 90-day mortality rate of LC patients in our cohort was still higher than in other studies. The Borgonovo study conducted in Brazil showed that the mortality of cirrhotic patients with acute decompensation was 35.3% [11]. The 90-day mortality rate for cirrhotic patients in this study was found to be higher compared to Berni et al’s study which reported a mortality rate of 23% [12]. The study by Maccali revealed that the 90-day mortality rate for hospitalized cirrhotic patients with an elevated neutrophil-to-lymphocyte ratio reached 38%, whereas patients without an increase in this ratio had a mortality rate of 17% [13]. The Maccali study revealed that the features of cirrhotic patients with Child-Pugh C nearly identical to those in this study (43.1%). However, the mortality rate in the Maccali study was slightly lower compared to Gani et al’s study (38% vs. 42.2%) [11, 13].

In this study, the majority of the patients had liver cirrhosis with decompensated conditions. Gastrointestinal bleeding is a serious complication associated with an increase in portal hypertension in patients with liver cirrhosis. Mild, moderate, and large esophageal varices are commonly found in patients with portal hypertension. The presence of significant esophageal varices is associated with advanced Child-Pugh’s condition. The study conducted at Cipto Mangunkusumo General Hospital by Kalista reported that 65.3% of patients with Child-Pugh B had large esophageal varices, while 85.6% of patients with Child-Pugh C had large esophageal varices [14]. An elevation in portal venous pressure can result in the rupture of esophageal varices, leading to gastrointestinal bleeding. The mortality rate of cirrhotic patients is increased by 7–15% due to gastrointestinal bleeding, which can be caused by esophageal or gastric varices [15]. In these patients, all patients with acute variceal bleeding underwent endoscopy within 12–24 h after bleeding. EVL was performed during the endoscopy procedure as primary prophylaxis, along with vasoactive medication, antibiotics, and blood transfusion.

Ascites is a common complication among patients with liver cirrhosis and is characterized by an abnormal accumulation of fluid in the abdomen. Those with severe ascites are at an increased risk of developing spontaneous bacterial peritonitis (SBP) due to immune system dysfunction or bacterial translocation [16]. The presence of ascites in patients with liver cirrhosis has been associated with an increased risk of mortality, with incidence rates of 15% within the first year and 44% within the first five years [17]. In our study cohort, patients with significant ascites underwent paracentesis. The fluid samples from paracentesis were then analyzed to detect the presence of pathogenic bacteria. For patients with positive cultures indicating spontaneous bacterial peritonitis (SBP), intravenous antibiotics were administered.

**Fig. 3:**
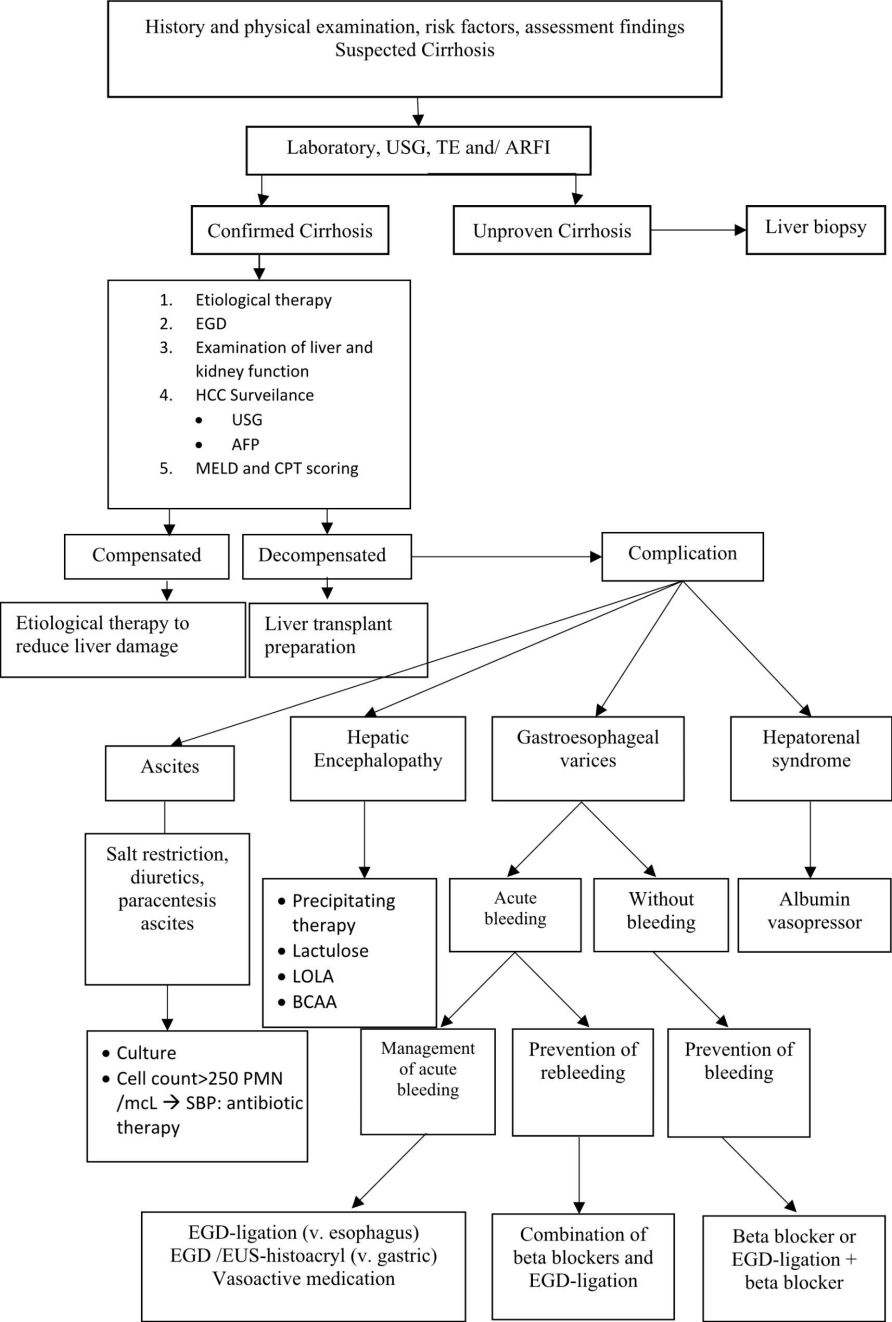
Algorithm for Diagnosis and Treatment of Cirrhosis based on clinical practice guidelines in Cipto Mangunkusumo Hospital

Patients with liver cirrhosis commonly experience neuropsychiatric complications, including hepatic encephalopathy. This condition typically arises in 30–45% of patients who have reached a decompensated state [18]. L-Ornithine L-Aspartate (LOLA), branched-chain amino acids (BCCA), lactuloses, and other supportive treatment were given according to the severity of hepatic encephalopathy. The standard protocol for managing cirrhosis complications at our hospital is described in Fig. 3.

### Predictors of mortality of liver cirrhosis patients in 90-Day post hospitalization at Cipto Mangunkusumo Hospital

In this study, the predictors of mortality in cirrhotic patients after 90 days of hospitalization were Child-Pugh, MELD, and leukocyte. The Child-Pugh scoring system is composed of several parameters, including serum bilirubin, serum albumin, prothrombin time, presence of ascites, and encephalopathy, which are utilized to forecast mortality risk among patients with liver cirrhosis [19]. This study found that the risk of death was 14.59 times higher in patients with Child-Pugh C compared to those with Child-Pugh A (HR = 14.59; 95% CI: 1.77–120.16, p = 0.013). The study conducted by Younis on patients with chronic liver disease and hepatopulmonary syndrome showed that an increase in Child-Pugh score was associated with a higher risk of death, with a hazard ratio (HR) of 1.64 (95% CI: 1.22–2.21, p < 0.01) [20]. Child-Pugh is a scoring system to determine the degree of liver damage in patients with liver cirrhosis that has been used universally to help predicting the risk of death and the development of complications due to liver dysfunction, such as gastrointestinal bleeding, development of ascites, jaundice, or encephalopathy. After the first onset of hepatic dysfunction complications, mortality significantly increases in patients with liver cirrhosis [19].

Another predictor found in this study was MELD. MELD was originally designed for screening liver transplant recipients, but is now widely used to predict mortality in cirrhotic patients. This study found that MELD above 14 increased the risk of death by 3.20 times higher than Child-Pugh A (HR = 3.20; 95% CI: 1.16–8.85, p = 0.001). In the Havens study involving 707 patients with chronic liver disease, those with MELD scores greater than 10 exhibited a 1.45-fold higher risk of death within 90 days of follow-up [21]. MELD scores are calculated in a more objective manner when compared to Child-Pugh. Numerous studies have validated its use, making it a widely accepted scoring system. Studies have consistently observed that cirrhotic patients with Child-Pugh C and MELD scores exceeding 14 have a higher incidence of morbidity and mortality [22, 23].

Another parameter in this study related to mortality in patients with liver cirrhosis is leukocytes. In this study, the risk of death was 2.83 times higher in liver cirrhosis patients with a leukocyte count above 12.00 than those with a count below this threshold (HR = 2.83; 95% CI: 1.54–5.17, p = 0.00). Similarly, the Bal CK et al. study in decompensated liver cirrhosis patients observed a higher mean leukocyte count in those who died compared to those who survived (15.17 vs. 11.86). Moreover, elevated leukocyte levels have been linked to in-hospital mortality caused by SBP infection and septic shock [24].

### Clinical application of Child-Pugh, MELD, and leukocyte scoring system for predicting mortality of liver cirrhosis patients in 90-day post hospitalization at Cipto Mangunkusumo Hospital

The purpose of this study was to develop a scoring system that categorizes inpatient cirrhosis patients based on their risk level and the urgency of intensive care needed. The final multivariate analysis identified three variables that predict 90-day mortality in cirrhotic patients: Child-Pugh, MELD, and leukocyte count. A diagnostic test was performed to assess the predictive value of these three variables in determining the mortality of cirrhotic patients. The combination of the three variables yielded an area under the curve (AUC) of 0.921 (95% CI: 0.876–0.967), with a sensitivity of 81.6% and a specificity of 85.1%. Another study by Wu et al. utilized a similar scoring system to our study, where they found that Child-Turcotte-Pugh and MELD scores could predict mortality in liver cirrhosis patients, with AUCs of 0.81 (95% CI: 0.77–0.84) and 0.78 (95% CI: 0.74–0.81), respectively. However, our study demonstrated a higher AUC by combining these two parameters along with leukocyte count. Therefore, the combination of these three parameters can be a reliable tool for predicting 90-day mortality in patients with liver cirrhosis [25].

This study identified three variables to be incorporated into a scoring system for patients with liver cirrhosis, which has a score range of 0 to 11. Patients with liver cirrhosis will receive a score of 5 if Child-Pugh C, 3 if MELD > 14, and 3 if leukocytes > 12,000. The patients were categorized into three risk groups based on their calculated score. By implementing this scoring system, it is recommended that patients classified as low risk (with a score of 0–3 and a probability of death ranging from 4.1 to 18.4%) can be treated in an inpatient room. When the risk of death increases to moderate risk (with a score of 5–6 and a probability of death ranging from 40.5 to 54.2%), intensive care in the ICU is recommended for liver cirrhosis patients. Meanwhile, liver transplantation should be considered an emergency priority for high-risk patients (with a score of 8–11 and a probability of death ranging from 78.1 to 94.9%). However, it is important to note that this scoring system may need to be adjusted on a case-by-case basis, according to bed capacity and ICU policy in each center.

Admission to ICU in patients with decompensated cirrhosis is required in cases of advanced liver disease with acute complications. However, not all patients with advanced clinical conditions and the severity of liver disease have better clinical outcomes even after receiving ICU treatment. The prognosis of patients with liver cirrhosis is influenced by the severity of liver disease and the worsening state of extra-hepatic organ function. Clinicians need to consider the limited availability of ICU beds and the high expense of ICU treatment to utilize the ICU room appropriately. A scoring system should be regarded as an adjunct rather than a substitute for clinical judgment in the decision process concerning whether a patient should be admitted to the ICU because there are no absolute criteria to predict which cirrhotic decompensated patients will improve with normalization of organ function or deteriorate progressively [26].

Currently, there are specific scoring system used in cirrhosis patients to assess their liver function, such as Child-Turcotte-Pugh (CTP) and Model for End-Stage Liver Disease (MELD). Additionally, there are other general scoring systems that can also be used in cirrhosis patients admitted to the ICU, such as Acute Physiology And Chronic Health Evaluation (APACHE) II and III, Sequential Organ Failure Assessment (SOFA), Multiple Organ Dysfunction Score (MODS), and RIFLE (Risk, Injury, Failure, Loss And End-Stage Renal Failure) [26]. The scoring system using CTP and MELD is useful in indicating the severity of liver disease, but it still has prognostic errors in predicting death caused by extrahepatic organ dysfunction. Kavli et al. reported in their study that an increase in the number of organ failures in patients with liver cirrhosis was associated with a higher mortality rate in alcoholic patients with liver cirrhosis [27]. Patients with three or more organ failure conditions had a mortality rate of over 90% while in the ICU. In a similar study of ICU patients with liver cirrhosis, Saliba et al. found that SOFA and APACHE II scores were superior to CTP and MELD scores, with mortality ranging from 34–69% [28].

### Limitation and strength

This research has limitations that should be taken into consideration. The results of this study need to be interpreted with caution as some patients were lost to follow-up. However, the characteristics of these patients did not differ significantly from those included in the final analysis based on Child-Pugh (p = 0.530), MELD (p = 0.178), and Leucocyte (p = 0.291). The potential for selection bias is minimal since the lost follow-up patients were similar to those included in the final analysis. Additionally, this is a single-center study with a small sample size, which means that the findings may not be generalizable to other countries or populations.

Despite the need for cautious interpretation of the results, this study provides a valuable contribution by validating a previous retrospective cohort study conducted by Gani et al in 2017. The reliability of Child Pugh and MELD as tools for assessing mortality in LC patients is supported by the findings of both studies, which were conducted in similar hospital settings.

## Conclusion

The 90-day mortality of patients with liver cirrhosis after hospitalization was 42.2%. The factors that contributed were Child-Pugh, MELD, and leukocytes. The discriminative value of the AUC from the combination of these three variables was promising, with an AUC of 0.921 (95% CI: 0.876–0.967). According to this scoring system, patients were categorized into three risk categories: low risk (score of 0–3, probability of patient’s death 4.1% − 18.4%), moderate risk (score of 5–6, probability of patient’s death 40.5% − 54.2%), and high risk (score of 8–11, probability of patient’s death 78.1% − 94.9%). Clinicians can use this scoring system upon admission to determine the clinical judgment of cirrhotic patients.

## Data Availability

The dataset is available from the corresponding author on reasonable request.

## Abbreviations

ALT: Alanine aminotransferase
APACHE: Acute Physiology And Chronic Health Evaluation
AST: Aspartate aminotransferase
AUC: Area under the receiver operating characteristic curve
BCCA: Branched-Chain Amino Acids
CI: Confidence Interval
CTP: Child–Turcotte–Pugh
EVL: Endoscopic Variceal Ligation
HBV: Hepatitis B Virus
HCV: Hepatitis C Virus
HR: Hazard Ratio
ICD-10: International Classification of Diseases 10th
ICU: Intensive Care Unit
LC: Liver Cirrhosis
LOLA: L-Ornithine L-Aspartate
MAFLD: Metabolic-associated Fatty Liver Disease
MELD: Model for End-stage Liver Disease
MODS: Multiple Organ Dysfunction Score
NASH: Nonalcoholic Steatohepatitis
NBNC: Non-B Non-C
RIFLE: Risk, Injury, Failure, Loss And End-Stage Renal Failure
SBP: Spontaneous Bacterial Peritonitis
SD: Standard Deviation
SOFA: Sequential Organ Failure Assessment.
